# The potential use of non-fungible tokens (NFTs) in healthcare and medical research

**DOI:** 10.1371/journal.pdig.0000312

**Published:** 2023-07-27

**Authors:** Antonio Yaghy, Nicole Rose I. Alberto, Isabelle Rose I. Alberto, Rene S. Bermea, Ljubica Ristovska, Maria Yaghy, Sandra Hoyek, Nimesh A. Patel, Leo Anthony Celi

**Affiliations:** 1 New England Eye Center, Tufts University Medical Center, Boston, Massachusetts, United States of America; 2 College of Medicine, University of the Philippines, Manila, Philippines; 3 Division of Pulmonary & Critical Care Medicine, Massachusetts General Hospital, Harvard Medical School, Boston, Massachusetts, United States of America; 4 Harvard University, Faculty of Arts and Sciences, Department of Economics, Cambridge, Massachusetts, United States of America; 5 Centre Hospitalier Universitaire Timone Enfants, Marseille, France; 6 Department of Ophthalmology, Massachusetts Eye and Ear, Harvard Medical School, Boston, Massachusetts, United States of America; 7 Department of Ophthalmology, Boston Children’s Hospital, Boston, Massachusetts, United States of America; 8 Laboratory for Computational Physiology, MIT Institute for Medical Engineering and Science, Massachusetts Institute of Technology, Cambridge, Massachusetts, United States of America; 9 Information Systems, Beth Israel Deaconess Medical Center, Boston, Massachusetts, United States of America; Suez Canal University, EGYPT

## Abstract

Non-fungible tokens (NFTs) are cryptographic assets recorded on the blockchain that can certify authenticity and ownership, and they can be used to monetize health data, optimize the process of receiving a hematopoietic stem cell transplant, and improve the distribution of solid organs for transplantation. Blockchain technology, including NFTs, provides equitable access to wealth, increases transparency, eliminates personal or institutional biases of intermediaries, reduces inefficiencies, and ensures accountability. Blockchain architecture is ideal for ensuring security and privacy while granting individuals jurisdiction over their own information, making it a unique solution to the current limitations of existing health information systems. NFTs can be used to give patients the option to monetize their health data and provide valuable data to researchers. Wearable technology companies can also give their customers the option to monetize their data while providing data necessary to improve their products. Additionally, the process of receiving a hematopoietic stem cell transplant and the distribution of solid organs for transplantation could benefit from the integration of NFTs into the allocation process. However, there are limitations to the technology, including high energy consumption and the need for regulatory guidance. Further research is necessary to fully understand the potential of NFTs in healthcare and how it can be integrated with existing health information technology. Overall, NFTs have the potential to revolutionize the healthcare sector, providing benefits such as improved access to health information and increased efficiency in the distribution of organs for transplantation.

## Introduction

Non-fungible tokens (NFTs) are cryptographic assets recorded on the blockchain. A blockchain is a digital ledger technology that securely and transparently records transactions across a decentralized network of computers, nodes, or servers, without the need for intermediaries. Unlike money, which is fungible, NFTs cannot be substituted, subdivided, or traded for equivalency. Their storage on an immutable blockchain provides certification of authenticity and ownership. Essentially, NFTs are digital certificates of ownership that can be bought and sold in online marketplaces. These unique features open doors for a myriad of uses in many sectors, including healthcare. Herein, we discuss blockchain and its use for promoting health equity, the potential applications of NFTs in healthcare, and the limitations of such technology.

## Blockchain and health equity

Blockchain is a decentralized immutable ledger that makes it easier to record transactions and track virtually any asset of value (including NFTs) [1]. This technology has the ability to provide more equitable access to wealth by increasing the accessibility of assets and transparency of transactions without having to go through traditional gatekeepers. Blockchain technology, including NFTs, has eliminated roadblocks to world markets, granting people from the poorest to the wealthiest access to a safe and secure transnational network [2]. It presents a unique opportunity for investors from different cultures and backgrounds to participate. Particularly, the NFT space has encouraged the representation of minorities, including women, BIPOC, and LGBTQ [3]. Similarly, blockchain technology also has huge potential for ushering in a new era of healthcare financing that will enable global health equity and universal health coverage [2]. Being built upon a trusted, secure, immutable, and interoperable network, blockchains can revolutionize health information technology and payment mechanisms, eliminating the personal or institutional biases of intermediaries on the nature and scope of transactions. Transactional inefficiencies are reduced by making cross-border payments cheaper, faster, and safer through secure direct financing schemes. Unlike traditional financial intermediaries, transactions made on the blockchain are easily verifiable, allowing full transparency and accountability. Smart contracts will enable results-based financing by utilizing unique identifiers for supplies, equipment, and patient care results, in combination with time and geolocation stamps. Furthermore, smart contracts have the capacity to ensure that funds intended for large capital purchases are delivered to recipients, avoiding fund mismanagement, fraud, and corruption [2]. Moreover, the architecture of blockchain technology is ideal for ensuring security and privacy while granting individuals jurisdiction over their own information. The cryptographic security and inability to change any entry make it a unique solution to the current limitations of existing health information systems. For example, blockchain technology has potential utility in addressing health inequity in people experiencing homelessness. The lack of proof of identity has become a major barrier to healthcare and social services in this population. Through blockchain technology, people experiencing homelessness can access secure and portable identity management [4]. The approach involves educating individuals who are experiencing homelessness about the advantages of utilizing blockchain technology to securely store and exchange their personal documents. Their input and opinions are taken into account before agreeing to participate, and if they opt-in, they can attend designated pop-up clinics that offer various health, social, and other support services [5].

## Potential applications of NFTs in healthcare and medical research

NFTs allow the tokenization of all types of data (e.g., text, images, video, and audio) and give individuals the option to monetize their data and receive royalties every time they are sold to a new buyer. When it comes to health data, NFTs have the capability of putting patients back in the driver’s seat as opposed to keeping health data vendors in total control of the fate and use of their data [6]. One potential use for NFTs in this regard is giving patients the option, at the time of signing informed consent, to mint health data collected when receiving care as an NFT. Minting is the method in which digital information is converted into a token on the blockchain. With NFTs, patients could potentially sell access to their health data to organizations that need it for research purposes. This could provide a new source of income for patients while also providing valuable data to researchers. The longer the duration of consent and the fewer restrictions patients give for the use of the data, the higher monetary value the NFT would carry. Likewise, wearable technology companies can give their customers the option, at the time of purchase, of minting an NFT that will determine the scope and duration, of the utilization of the collected data, thus granting patients the option to monetize their data while providing data necessary to improve their products.

Moreover, NFTs can help optimize the process of receiving a hematopoietic stem cell transplant [7]. Booth and Gehrie suggested that this can be done by having stem cell donor centers enter information about HLA and ABO blood typing as well as transplant product availability directly into NFTs and make it publicly available for transplant centers to access. This allows transplant centers that would traditionally order stem cells from donor centers based on a prioritization list to see exactly what transplant products are available and hence match their needs. This more efficient inventory management system based on real-time need potentially reduces donor center requirements for excessive stock of cryopreserved products [7]. Similarly, the process of distribution of solid organs from deceased donors for transplantation is incredibly complex and could benefit from the integration of NFTs into the allocation process. Progress in the areas of deceased donor management and factors relating to organ offer decisions has been stunted by a lack of both transplant center communication and transparency [8,9]. Blockchain provides a decentralized, secure, and transparent platform for storing data. This makes it possible for both transplant centers and donor centers to openly share information in a way that is both tamper-proof and easily accessible. Additionally, NFTs use smart contracts, self-executing agreements with the terms of the contract directly written into code. This allows for automated and efficient management of data, reducing the risk of errors and miscommunication. This level of information availability could drive discovery, facilitate more efficient transplants, increase accountability, and build the public’s trust in the organ transplantation process. Importantly, NFTs allow certain data to be unlocked at the time of transfer, hence the ability to store sensitive information securely and display only what needs to be shared publicly.

When it comes to research, NFTs can offer protection of intellectual property [10]. For example, researchers who have a novel idea or hypothesis that is worth testing, but lack the resources or facilities to conduct this research, can create an NFT and share their idea publicly. Given the immutable nature of the blockchain, a timestamp at the time of NFT minting would protect this researcher’s idea from potential theft. Hence, the researcher has the option to sell the idea for a certain price that they believe the idea is worth. This could lead to a revolution in medical research as hundreds of potentially ground-breaking ideas are lost every year without being shared with the scientific community because of the lack of enough platforms to share these ideas or the lack of an economic incentive to do so. Another potential use is to incentivize peer reviewers of open-access journals to conduct a peer review on time and reward them fairly for their efforts [11]. For example, peer-reviewed journals could reward peer reviewers, at the time of review completion, with NFTs that can range from publication fee waivers to travel support to attend academic conferences based on the number of articles reviewed per year. The main advantage of minting these rewards as NFTs is the seamless transferability of rewards between journals due to the tradable nature of NFTs. This allows reviewers to exchange or sell their reward received from one journal in an exchange for credit in another journal through interacting with other reviewers active in that particular NFT marketplace.

## Limitations

As with any nascent technology, NFTs have raised several concerns [12], mainly regarding their carbon footprint, as the technology is dependent on computational-heavy tasks that validate transactions on the blockchain [13]. However, new cryptographic technologies carrying a significantly lower carbon footprint will be implemented in the near future while allowing the evolution of the technology with time [14]. In addition, because NFTs hold certain monetary value and are listed online, these can attract unethical players that will try to steal those assets from less technologically savvy people. Since NFTs can contain patient health information, they may need to be regulated in terms of storage on the blockchain and exchange on marketplaces to ensure compliance with the Health Insurance Portability and Accountability Act (HIPAA) and similar regulatory entities abroad [15].

In addition, the adoption of NFT technology in healthcare may face resistance from data vendors and insurance companies as it may result in a decrease in profit margins associated with the use of health data. However, in the era of big data and machine learning, NFTs for data can address the ethical concerns surrounding the use of publicly available patient data for AI algorithm development and monetization without providing financial compensation to the patients [6].

The potential disadvantage of using NFTs as assets is the exacerbation of wealth disparities, where entities with more financial resources can acquire digital assets at the expense of smaller entities, thus validating the old adage “the rich get richer and the poor get poorer” [16]. This could lead to a situation where a better-funded institution in a developed country acquires an NFT created by a researcher in a developing country and conducts research that may not be representative of or benefit the population in the latter. To address this issue, solutions such as limiting the number of NFTs that an institution can purchase or increasing awareness among NFT sellers to consider the impact of their sales on wealth disparities can be implemented. Another solution is for sellers to impose conditions, such as the inclusion of minority groups in any research studies, at the time of NFT listing.

## Conclusions

NFT technology is still in its infancy, and its extensive potential to improve healthcare delivery in the upcoming years remains to be explored. NFTs have a similar potential to revolutionize many sectors, including healthcare and medical research. Their unique features of being digital and non-fungible allow the tokenization and monetization of health data. Their storage on an immutable blockchain allows the democratization of the technology, making it available worldwide and eliminating roadblocks to current global markets. However, raising awareness of this technology and regulating NFT marketplaces that contain health data will be necessary to expand this technology and prevent malicious exploitation.
